# Trimethyl­ammonium 2,6-dioxo-5-(2,4,6-trinitro­phen­yl)-1,2,3,6-tetra­hydro­pyrimidin-4-olate

**DOI:** 10.1107/S1600536811051610

**Published:** 2011-12-07

**Authors:** D. Kalaivani, M. Buvaneswari, S. Rajeswari

**Affiliations:** a PG and Research Department of Chemistry, Seethalakshmi Ramaswami College, Tiruchirappalli 620 002, Tamil Nadu, India; b Department of Chemistry, Faculty of Engineering and Technology, SRM University, Kattankulathur 603 203, Tamil Nadu, India

## Abstract

In the title barbiturate salt (trivial name: trimethyl­ammonium 2,4,6-trinitro­phenyl­barbiturate), C_3_H_10_N^+^·C_10_H_4_N_5_O_9_^−^, the asymmetric unit contains two sets of anion–cation moieties. The dihedral angle between the rings in the anions are 44.0 (3) and 45.7 (3)°. Adjacent anions are connected into ribbons along [100] through *R*_2_^2^(8) ring motifs formed by N—H⋯O hydrogen bonds involving the barbiturate rings. Attached to both sides of these ribbons *via* N—H⋯O hydrogen bonds are the trimethyl­ammonium cations. C—H⋯O hydrogen bonds are also observed.

## Related literature

For the biological activity of barbiturates, see: Nogrady (1988 ▶); Gitto *et al.* (2006 ▶). For side effects of barbiturates, see: Hardman & Limbird (2001 ▶); Rana *et al.* (2007 ▶). For barbit­ur­ates related to the title compound, see: Kalaivani *et al.* (2008 ▶); Kalaivani & Malarvizhi (2009 ▶).
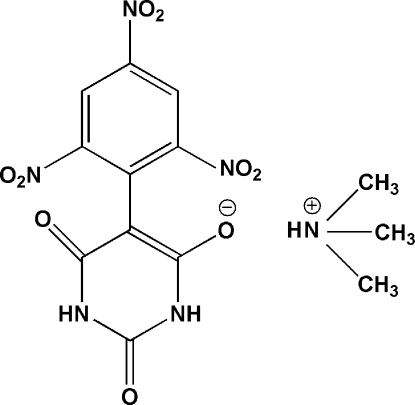


## Experimental

### 

#### Crystal data

C_3_H_10_N^+^·C_10_H_4_N_5_O_9_^−^
*M* *_r_* = 398.30
Monoclinic, 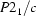

*a* = 11.9828 (12) Å
*b* = 30.802 (3) Å
*c* = 9.5516 (11) Å
β = 105.895 (6)°
*V* = 3390.6 (6) Å^3^
*Z* = 8
Mo *K*α radiation
μ = 0.13 mm^−1^
*T* = 296 K
0.30 × 0.20 × 0.20 mm

#### Data collection

Bruker Kappa APEX II CCD diffractometer
Absorption correction: multi-scan (*SADABS*; Bruker, 2004 ▶) *T*_min_ = 0.90, *T*_max_ = 0.97
31796 measured reflections
6580 independent reflections
4977 reflections with *I* > 2σ(*I*)
*R*_int_ = 0.030

#### Refinement

*R*[*F* ^2^ > 2σ(*F* ^2^)] = 0.046
*wR*(*F* ^2^) = 0.123
*S* = 1.02
6580 reflections
536 parameters
H atoms treated by a mixture of independent and constrained refinement
Δρ_max_ = 0.34 e Å^−3^
Δρ_min_ = −0.30 e Å^−3^

### 

Data collection: *APEX2* (Bruker, 2004 ▶); cell refinement: *SAINT-Plus* (Bruker, 2004 ▶); data reduction: *SAINT-Plus*; program(s) used to solve structure: *SIR92* (Altomare *et al.*, 1993 ▶); program(s) used to refine structure: *SHELXL97* (Sheldrick, 2008 ▶); molecular graphics: *ORTEP-3* (Farrugia, 1997 ▶) and *Mercury* (Macrae *et al.*, 2008 ▶); software used to prepare material for publication: *SHELXL97*.

## Supplementary Material

Crystal structure: contains datablock(s) global, I. DOI: 10.1107/S1600536811051610/qk2022sup1.cif

Structure factors: contains datablock(s) I. DOI: 10.1107/S1600536811051610/qk2022Isup2.hkl

Supplementary material file. DOI: 10.1107/S1600536811051610/qk2022Isup3.cml

Additional supplementary materials:  crystallographic information; 3D view; checkCIF report

## Figures and Tables

**Table 1:** Hydrogen-bond geometry (Å, °)

*D*—H⋯*A*	*D*—H	H⋯*A*	*D*⋯*A*	*D*—H⋯*A*
N4—H4⋯O17^i^	0.85 (2)	1.94 (2)	2.780 (2)	170 (2)
N5—H5⋯O16	0.86 (3)	1.93 (3)	2.778 (2)	170 (2)
N9—H9⋯O8	0.85 (2)	1.95 (2)	2.790 (2)	172 (2)
N10—H10⋯O7^ii^	0.83 (2)	1.96 (2)	2.786 (2)	173 (2)
N11—H11*A*⋯O9^iii^	0.94 (3)	1.82 (3)	2.730 (2)	160 (3)
N12—H12*A*⋯O18	0.94 (3)	2.02 (3)	2.803 (3)	139 (3)
N12—H12*A*⋯O5^iv^	0.94 (3)	2.16 (3)	2.856 (3)	130 (2)
C2—H2⋯O4^v^	0.93	2.58	3.265 (3)	131
C16—H16⋯O2^vi^	0.93	2.27	3.146 (3)	156
C24—H24*B*⋯O5	0.96	2.51	3.448 (4)	164
C24—H24*C*⋯O10^iii^	0.96	2.57	3.457 (4)	154
C26—H26*B*⋯O18^iv^	0.96	2.50	3.400 (5)	156

Symmetry codes: (i) 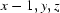; (ii) 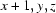; (iii) 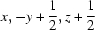; (iv) 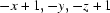; (v) 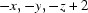; (vi) 
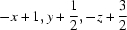
.

**Fig. 1.**
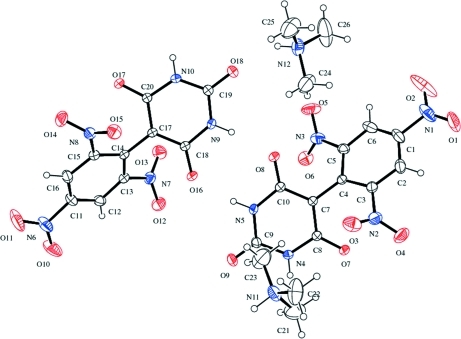
ORTEP diagram of (I) showing 30% probability displacement ellipsoids and the atomic numbering scheme.

**Fig. 2.**
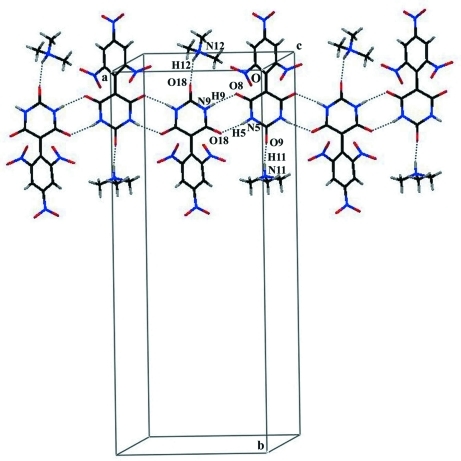
View of a hydrogen bonded ribbon of barbiturate anions with the attached trimethylammonium cations.

## supplementary crystallographic information

### Comment

Barbiturates are biologically active molecules (Nogrady, 1988; Gitto *et al.*, 2006). Many reported barbiturates have noticeable side effects (Hardman & Limbird, 2001; Rana *et al.*, 2007). The growing interest in preparing new barbiturates prompted the present work. A barbiturate previously synthesized in our laboratory from 1-chloro-2,4-dinitrobenzene, barbituric acid and triethylamine has only one anion-cation pair in the asymmetric unit (Kalaivani *et al.*, 2008; Kalaivani & Malarvizhi, 2009). However, the asymmetric unit of the title barbiturate (Scheme), prepared from 1-chloro-2,4,6-trinitrobenzene, barbituric acid and trimethylamine, comprises two sets of anion-cation moieties (Fig. 1). Two sets of *R*_2_^2^(8) hydrogen bond motifs are noticed between the adjacent anions. The hydrogen bonds N9—H9···O8 and N5—H5···O16 form the first set of *R*_2_^2^(8) motif and the hydrogen bonds N10—H10···O7 and N4—H4···O17 form the second set of *R*_2_^2^(8) motif. The aggregations of these motifs form an infinite one dimensional anionic ribbon extending along [100] direction. To either side of the anionic ribbon the trimethylammonium cations are hydrogen bonded through N11—H11A···O9 and N12—H12A···O18 hydrogen bonds in the terminal positions as depicted in Fig. 2. Furthermore a number of weak C—H···O interactions (cf. Table 1) contribute to the three-dimensional coherence of the crystal structure.

### Experimental

1-Chloro-2,4,6-trinitrobenzene (2.5 g, 0.01 mol) in 20 ml of absolute ethanol was mixed with barbituric acid (1.3 g, 0.01 mol) in 30 ml of absolute ethanol and heated to 40°C. Trimethylamine (3 ml ~0.05 mol) was then added and the mixture was shaken well for 2 hrs. Dark maroon red crystals obtained were filtered. The filtered crystals were powdered well and washed with 50 ml of dry ether and recrystallized from absolute ethanol (yield 80%; m.p. 245 °C). Good quality crystals, suitable for single-crystal X-ray studies were obtained by slow evaporation of the ethanolic solution of title compound at room temperature.

### Refinement

The six N-bound H atoms were located in a difference map and were freely refined without constraints. The C-bound hydrogen atoms were placed in calculated positions (C—H = 0.93–0.96 Å) and refined as riding atoms with *U*_iso_(H) = 1.2*U*_eq_(C) for non-methyl and *U*_iso_(H) = 1.5*U*_eq_(C) for methyl H.

### Crystal data

**Table:**

C_3_H_10_N^+^·C_10_H_4_N_5_O_9_^−^	*F*(000) = 1648
*M**_r_* = 398.30	*D*_x_ = 1.561 Mg m^−^^3^
Monoclinic, *P*2_1_/*c*	Mo *K*α radiation, λ = 0.71073 Å
Hall symbol: -P 2ybc	Cell parameters from 8488 reflections
*a* = 11.9828 (12) Å	θ = 2.2–29.2°
*b* = 30.802 (3) Å	µ = 0.13 mm^−^^1^
*c* = 9.5516 (11) Å	*T* = 296 K
β = 105.895 (6)°	Block, brown
*V* = 3390.6 (6) Å^3^	0.30 × 0.20 × 0.20 mm
*Z* = 8	

### Data collection

**Table:**

Bruker Kappa APEX II CCD diffractometer	6580 independent reflections
Radiation source: fine-focus sealed tube	4977 reflections with *I* > 2σ(*I*)
graphite	*R*_int_ = 0.030
ω and φ scan	θ_max_ = 26.0°, θ_min_ = 2.2°
Absorption correction: multi-scan (*SADABS*; Bruker, 2004)	*h* = −14→14
*T*_min_ = 0.90, *T*_max_ = 0.97	*k* = −37→37
31796 measured reflections	*l* = −11→11

### Refinement

**Table:**

Refinement on *F*^2^	Secondary atom site location: difference Fourier map
Least-squares matrix: full	Hydrogen site location: inferred from neighbouring sites
*R*[*F*^2^ > 2σ(*F*^2^)] = 0.046	H atoms treated by a mixture of independent and constrained refinement
*wR*(*F*^2^) = 0.123	*w* = 1/[σ^2^(*F*_o_^2^) + (0.0538*P*)^2^ + 1.671*P*] where *P* = (*F*_o_^2^ + 2*F*_c_^2^)/3
*S* = 1.02	(Δ/σ)_max_ < 0.001
6580 reflections	Δρ_max_ = 0.34 e Å^−^^3^
536 parameters	Δρ_min_ = −0.30 e Å^−^^3^
0 restraints	Extinction correction: *SHELXL97* (Sheldrick, 2008), Fc^*^=kFc[1+0.001xFc^2^λ^3^/sin(2θ)]^-1/4^
Primary atom site location: structure-invariant direct methods	Extinction coefficient: 0.0021 (4)

### Special details

**Table:**

Geometry. All esds (except the esd in the dihedral angle between two l.s. planes) are estimated using the full covariance matrix. The cell esds are taken into account individually in the estimation of esds in distances, angles and torsion angles; correlations between esds in cell parameters are only used when they are defined by crystal symmetry. An approximate (isotropic) treatment of cell esds is used for estimating esds involving l.s. planes.
Refinement. Refinement of F^2^ against ALL reflections. The weighted R-factor wR and goodness of fit S are based on F^2^, conventional R-factors R are based on F, with F set to zero for negative F^2^. The threshold expression of F^2^ > 2sigma(F^2^) is used only for calculating R-factors(gt) etc. and is not relevant to the choice of reflections for refinement. R-factors based on F^2^ are statistically about twice as large as those based on F, and R- factors based on ALL data will be even larger.

### Fractional atomic coordinates and isotropic or equivalent isotropic displacement parameters (Å^2^)

**Table:**

	*x*	*y*	*z*	*U*_iso_*/*U*_eq_	
C1	0.1280 (2)	−0.04978 (7)	0.6975 (3)	0.0520 (6)	
C2	0.07387 (19)	−0.02594 (7)	0.7794 (3)	0.0507 (6)	
H2	0.0396	−0.0393	0.8444	0.061*	
C3	0.07113 (16)	0.01807 (6)	0.7636 (2)	0.0379 (5)	
C4	0.11767 (14)	0.04061 (6)	0.6661 (2)	0.0301 (4)	
C5	0.16732 (16)	0.01315 (6)	0.5837 (2)	0.0371 (4)	
C6	0.17509 (19)	−0.03096 (7)	0.5989 (3)	0.0492 (6)	
H6	0.2116	−0.0476	0.5431	0.059*	
C7	0.11546 (15)	0.08708 (6)	0.6514 (2)	0.0294 (4)	
C8	0.01360 (15)	0.11016 (6)	0.6479 (2)	0.0284 (4)	
C9	0.10703 (16)	0.17708 (6)	0.6088 (2)	0.0330 (4)	
C10	0.21401 (15)	0.10922 (6)	0.6360 (2)	0.0314 (4)	
C11	0.57418 (19)	0.33262 (6)	0.5258 (2)	0.0418 (5)	
C12	0.49039 (18)	0.30675 (6)	0.4401 (2)	0.0386 (5)	
H12	0.4260	0.3186	0.3735	0.046*	
C13	0.50449 (16)	0.26296 (6)	0.4557 (2)	0.0322 (4)	
C14	0.59422 (15)	0.24254 (6)	0.5597 (2)	0.0288 (4)	
C15	0.67477 (16)	0.27170 (6)	0.6415 (2)	0.0321 (4)	
C16	0.66866 (19)	0.31572 (6)	0.6246 (2)	0.0417 (5)	
H16	0.7272	0.3336	0.6788	0.050*	
C17	0.60067 (15)	0.19623 (6)	0.5822 (2)	0.0303 (4)	
C18	0.50129 (15)	0.17370 (6)	0.5898 (2)	0.0314 (4)	
C19	0.61320 (16)	0.10691 (6)	0.6373 (2)	0.0372 (5)	
C20	0.70684 (16)	0.17435 (6)	0.6027 (2)	0.0326 (4)	
C21	−0.0029 (3)	0.19000 (12)	1.0012 (4)	0.0901 (10)	
H21A	−0.0174	0.1597	0.9804	0.135*	
H21B	−0.0129	0.2057	0.9117	0.135*	
H21C	−0.0563	0.2008	1.0515	0.135*	
C22	0.1314 (3)	0.17431 (12)	1.2299 (4)	0.0930 (11)	
H22A	0.1229	0.1435	1.2147	0.140*	
H22B	0.0744	0.1845	1.2757	0.140*	
H22C	0.2077	0.1805	1.2913	0.140*	
C23	0.1971 (3)	0.18289 (12)	1.0133 (4)	0.0934 (11)	
H23A	0.2747	0.1878	1.0727	0.140*	
H23B	0.1837	0.1997	0.9255	0.140*	
H23C	0.1871	0.1526	0.9892	0.140*	
C24	0.4800 (3)	0.00605 (12)	0.8010 (4)	0.0884 (10)	
H24A	0.4493	−0.0136	0.8593	0.133*	
H24B	0.4214	0.0122	0.7123	0.133*	
H24C	0.5033	0.0325	0.8538	0.133*	
C25	0.6768 (3)	−0.01717 (14)	0.8925 (4)	0.0988 (12)	
H25A	0.6566	−0.0353	0.9635	0.148*	
H25B	0.6975	0.0112	0.9329	0.148*	
H25C	0.7414	−0.0297	0.8656	0.148*	
C26	0.5432 (5)	−0.05449 (12)	0.6919 (5)	0.137 (2)	
H26A	0.6101	−0.0716	0.6914	0.205*	
H26B	0.4992	−0.0487	0.5935	0.205*	
H26C	0.4959	−0.0702	0.7411	0.205*	
N1	0.1341 (2)	−0.09686 (8)	0.7141 (3)	0.0774 (8)	
N2	0.02277 (18)	0.04092 (7)	0.8676 (2)	0.0520 (5)	
N3	0.20994 (17)	0.02933 (6)	0.4651 (2)	0.0493 (5)	
N4	0.01520 (13)	0.15431 (5)	0.62608 (18)	0.0327 (4)	
N5	0.20410 (14)	0.15362 (5)	0.61741 (19)	0.0352 (4)	
N6	0.5592 (2)	0.37934 (6)	0.5148 (2)	0.0626 (6)	
N7	0.42027 (15)	0.23778 (6)	0.34613 (19)	0.0409 (4)	
N8	0.76942 (14)	0.25697 (5)	0.7650 (2)	0.0396 (4)	
N9	0.51370 (14)	0.12959 (5)	0.6177 (2)	0.0383 (4)	
N10	0.70604 (14)	0.13030 (5)	0.6297 (2)	0.0380 (4)	
N11	0.11542 (19)	0.19574 (6)	1.0921 (2)	0.0538 (5)	
N12	0.5802 (2)	−0.01380 (7)	0.7670 (2)	0.0569 (5)	
O1	0.0869 (2)	−0.11310 (7)	0.7939 (5)	0.1399 (14)	
O2	0.1908 (3)	−0.11639 (7)	0.6489 (2)	0.1265 (13)	
O3	0.08047 (18)	0.06930 (6)	0.9405 (2)	0.0659 (5)	
O4	−0.06914 (17)	0.02796 (7)	0.8806 (2)	0.0788 (6)	
O5	0.29880 (18)	0.01269 (7)	0.4523 (2)	0.0780 (6)	
O6	0.15519 (15)	0.05664 (6)	0.38352 (18)	0.0575 (4)	
O7	−0.07663 (10)	0.09318 (4)	0.65984 (16)	0.0365 (3)	
O8	0.30776 (11)	0.09178 (4)	0.64047 (17)	0.0423 (4)	
O9	0.10310 (12)	0.21575 (4)	0.58780 (19)	0.0506 (4)	
O10	0.4647 (2)	0.39359 (6)	0.4567 (3)	0.0874 (7)	
O11	0.6406 (2)	0.40236 (6)	0.5663 (3)	0.1089 (9)	
O12	0.32075 (13)	0.25037 (6)	0.3120 (2)	0.0589 (5)	
O13	0.45480 (14)	0.20692 (5)	0.29116 (18)	0.0547 (4)	
O14	0.86373 (13)	0.27312 (5)	0.7787 (2)	0.0605 (5)	
O15	0.74674 (14)	0.23106 (5)	0.84766 (17)	0.0493 (4)	
O16	0.40506 (11)	0.19019 (4)	0.57728 (17)	0.0403 (3)	
O17	0.79831 (11)	0.19122 (4)	0.59685 (18)	0.0438 (4)	
O18	0.61782 (13)	0.06785 (5)	0.6586 (2)	0.0563 (5)	
H4	−0.046 (2)	0.1686 (7)	0.619 (2)	0.037 (6)*	
H5	0.263 (2)	0.1680 (8)	0.607 (3)	0.050 (7)*	
H9	0.455 (2)	0.1158 (7)	0.627 (2)	0.042 (6)*	
H10	0.769 (2)	0.1172 (7)	0.640 (2)	0.046 (6)*	
H11A	0.123 (3)	0.2258 (11)	1.111 (3)	0.091 (10)*	
H12A	0.605 (3)	0.0039 (10)	0.701 (3)	0.082 (9)*	

### Atomic displacement parameters (Å^2^)

**Table:**

	*U*^11^	*U*^22^	*U*^33^	*U*^12^	*U*^13^	*U*^23^
C1	0.0435 (12)	0.0251 (11)	0.0709 (16)	−0.0001 (9)	−0.0121 (11)	0.0031 (10)
C2	0.0354 (11)	0.0391 (12)	0.0711 (16)	−0.0048 (9)	0.0038 (11)	0.0197 (11)
C3	0.0274 (9)	0.0334 (10)	0.0516 (12)	0.0023 (8)	0.0089 (9)	0.0086 (9)
C4	0.0186 (8)	0.0273 (9)	0.0423 (11)	−0.0002 (7)	0.0046 (7)	0.0010 (8)
C5	0.0285 (10)	0.0337 (10)	0.0459 (12)	0.0032 (8)	0.0049 (8)	−0.0036 (9)
C6	0.0440 (12)	0.0348 (12)	0.0580 (14)	0.0105 (9)	−0.0043 (10)	−0.0105 (10)
C7	0.0226 (9)	0.0255 (9)	0.0406 (11)	−0.0002 (7)	0.0095 (7)	0.0016 (8)
C8	0.0240 (9)	0.0277 (9)	0.0336 (10)	−0.0004 (7)	0.0081 (7)	0.0008 (7)
C9	0.0278 (9)	0.0263 (10)	0.0427 (11)	−0.0017 (7)	0.0059 (8)	−0.0004 (8)
C10	0.0245 (9)	0.0288 (10)	0.0408 (11)	−0.0003 (7)	0.0089 (8)	0.0009 (8)
C11	0.0509 (12)	0.0252 (10)	0.0488 (13)	0.0038 (9)	0.0130 (10)	0.0035 (9)
C12	0.0382 (11)	0.0354 (11)	0.0426 (12)	0.0077 (8)	0.0118 (9)	0.0050 (9)
C13	0.0265 (9)	0.0338 (10)	0.0384 (11)	−0.0005 (8)	0.0123 (8)	−0.0006 (8)
C14	0.0233 (9)	0.0283 (9)	0.0389 (10)	0.0007 (7)	0.0153 (8)	−0.0004 (8)
C15	0.0274 (9)	0.0295 (10)	0.0400 (11)	0.0016 (7)	0.0103 (8)	0.0011 (8)
C16	0.0427 (11)	0.0287 (10)	0.0512 (13)	−0.0056 (9)	0.0086 (10)	−0.0027 (9)
C17	0.0236 (9)	0.0265 (9)	0.0432 (11)	0.0007 (7)	0.0133 (8)	−0.0007 (8)
C18	0.0248 (9)	0.0280 (9)	0.0436 (11)	0.0008 (7)	0.0131 (8)	−0.0007 (8)
C19	0.0317 (10)	0.0266 (10)	0.0558 (13)	−0.0001 (8)	0.0163 (9)	−0.0003 (9)
C20	0.0269 (9)	0.0252 (9)	0.0493 (12)	−0.0001 (7)	0.0166 (8)	−0.0011 (8)
C21	0.077 (2)	0.102 (3)	0.090 (2)	0.0316 (19)	0.0213 (18)	0.024 (2)
C22	0.103 (3)	0.100 (3)	0.068 (2)	−0.043 (2)	0.0111 (18)	0.0178 (18)
C23	0.082 (2)	0.116 (3)	0.091 (2)	−0.029 (2)	0.0376 (19)	−0.039 (2)
C24	0.0619 (18)	0.112 (3)	0.096 (2)	−0.0006 (18)	0.0298 (17)	0.028 (2)
C25	0.0619 (19)	0.140 (3)	0.087 (2)	0.003 (2)	0.0083 (17)	0.040 (2)
C26	0.227 (6)	0.081 (3)	0.130 (4)	−0.076 (3)	0.098 (4)	−0.039 (2)
N1	0.0761 (17)	0.0366 (13)	0.0909 (19)	0.0052 (12)	−0.0255 (14)	0.0109 (13)
N2	0.0495 (12)	0.0541 (12)	0.0587 (13)	0.0159 (10)	0.0256 (10)	0.0248 (10)
N3	0.0479 (11)	0.0517 (11)	0.0497 (11)	0.0081 (9)	0.0159 (9)	−0.0082 (9)
N4	0.0220 (8)	0.0269 (8)	0.0493 (10)	0.0040 (6)	0.0099 (7)	0.0017 (7)
N5	0.0226 (8)	0.0275 (8)	0.0561 (11)	−0.0036 (6)	0.0118 (7)	0.0042 (7)
N6	0.0797 (16)	0.0325 (11)	0.0665 (14)	0.0076 (11)	0.0046 (12)	0.0024 (10)
N7	0.0344 (9)	0.0435 (10)	0.0429 (10)	−0.0040 (8)	0.0071 (8)	0.0002 (8)
N8	0.0327 (9)	0.0342 (9)	0.0491 (11)	0.0004 (7)	0.0064 (8)	−0.0017 (8)
N9	0.0260 (8)	0.0278 (9)	0.0655 (12)	−0.0035 (7)	0.0201 (8)	0.0033 (8)
N10	0.0246 (8)	0.0279 (9)	0.0656 (12)	0.0049 (7)	0.0190 (8)	0.0027 (8)
N11	0.0733 (14)	0.0327 (10)	0.0583 (13)	−0.0046 (9)	0.0231 (11)	−0.0020 (9)
N12	0.0711 (14)	0.0527 (12)	0.0529 (12)	−0.0156 (10)	0.0272 (11)	0.0002 (10)
O1	0.0936 (19)	0.0485 (14)	0.275 (4)	−0.0012 (12)	0.047 (2)	0.064 (2)
O2	0.250 (4)	0.0466 (12)	0.0588 (13)	0.0597 (18)	0.0027 (17)	0.0002 (10)
O3	0.0789 (13)	0.0684 (12)	0.0515 (11)	0.0184 (11)	0.0197 (9)	0.0022 (9)
O4	0.0603 (11)	0.0869 (14)	0.1081 (16)	0.0190 (10)	0.0548 (11)	0.0474 (12)
O5	0.0752 (13)	0.0907 (14)	0.0825 (14)	0.0325 (11)	0.0459 (11)	−0.0048 (11)
O6	0.0557 (10)	0.0671 (11)	0.0479 (10)	0.0038 (9)	0.0113 (8)	0.0038 (8)
O7	0.0227 (6)	0.0326 (7)	0.0565 (9)	0.0023 (5)	0.0148 (6)	0.0072 (6)
O8	0.0217 (7)	0.0344 (8)	0.0731 (10)	0.0015 (5)	0.0169 (6)	0.0040 (7)
O9	0.0389 (8)	0.0248 (7)	0.0857 (12)	−0.0013 (6)	0.0131 (8)	0.0057 (7)
O10	0.1006 (17)	0.0439 (11)	0.0996 (16)	0.0302 (11)	−0.0035 (13)	0.0065 (10)
O11	0.1089 (18)	0.0312 (10)	0.151 (2)	−0.0119 (11)	−0.0244 (16)	0.0059 (12)
O12	0.0320 (8)	0.0625 (11)	0.0721 (11)	0.0003 (7)	−0.0030 (8)	0.0016 (9)
O13	0.0582 (10)	0.0518 (10)	0.0515 (10)	−0.0026 (8)	0.0108 (8)	−0.0162 (8)
O14	0.0315 (8)	0.0539 (10)	0.0856 (13)	−0.0096 (7)	−0.0015 (8)	0.0053 (9)
O15	0.0519 (9)	0.0494 (9)	0.0450 (9)	0.0027 (7)	0.0102 (7)	0.0097 (7)
O16	0.0236 (7)	0.0332 (7)	0.0684 (10)	0.0024 (5)	0.0197 (6)	0.0052 (7)
O17	0.0264 (7)	0.0306 (7)	0.0810 (11)	0.0005 (6)	0.0261 (7)	0.0031 (7)
O18	0.0430 (9)	0.0255 (8)	0.1043 (14)	0.0015 (6)	0.0269 (9)	0.0071 (8)

### Geometric parameters (Å, °)

**Table:**

C1—C6	1.354 (4)	C21—N11	1.456 (4)
C1—C2	1.360 (4)	C21—H21A	0.9600
C1—N1	1.458 (3)	C21—H21B	0.9600
C2—C3	1.363 (3)	C21—H21C	0.9600
C2—H2	0.9300	C22—N11	1.438 (4)
C3—C4	1.395 (3)	C22—H22A	0.9600
C3—N2	1.460 (3)	C22—H22B	0.9600
C4—C5	1.395 (3)	C22—H22C	0.9600
C4—C7	1.438 (3)	C23—N11	1.443 (4)
C5—C6	1.367 (3)	C23—H23A	0.9600
C5—N3	1.452 (3)	C23—H23B	0.9600
C6—H6	0.9300	C23—H23C	0.9600
C7—C8	1.405 (2)	C24—N12	1.461 (4)
C7—C10	1.405 (2)	C24—H24A	0.9600
C8—O7	1.234 (2)	C24—H24B	0.9600
C8—N4	1.377 (2)	C24—H24C	0.9600
C9—O9	1.207 (2)	C25—N12	1.424 (4)
C9—N4	1.352 (2)	C25—H25A	0.9600
C9—N5	1.352 (2)	C25—H25B	0.9600
C10—O8	1.235 (2)	C25—H25C	0.9600
C10—N5	1.380 (2)	C26—N12	1.452 (4)
C11—C16	1.363 (3)	C26—H26A	0.9600
C11—C12	1.365 (3)	C26—H26B	0.9600
C11—N6	1.451 (3)	C26—H26C	0.9600
C12—C13	1.362 (3)	N1—O1	1.178 (4)
C12—H12	0.9300	N1—O2	1.201 (4)
C13—C14	1.398 (3)	N2—O4	1.209 (3)
C13—N7	1.462 (3)	N2—O3	1.210 (3)
C14—C15	1.391 (3)	N3—O6	1.210 (2)
C14—C17	1.441 (2)	N3—O5	1.218 (2)
C15—C16	1.365 (3)	N4—H4	0.85 (2)
C15—N8	1.466 (3)	N5—H5	0.86 (3)
C16—H16	0.9300	N6—O11	1.198 (3)
C17—C18	1.398 (2)	N6—O10	1.199 (3)
C17—C20	1.405 (2)	N7—O12	1.211 (2)
C18—O16	1.235 (2)	N7—O13	1.212 (2)
C18—N9	1.384 (2)	N8—O15	1.205 (2)
C19—O18	1.219 (2)	N8—O14	1.208 (2)
C19—N10	1.344 (2)	N9—H9	0.85 (2)
C19—N9	1.350 (2)	N10—H10	0.83 (2)
C20—O17	1.228 (2)	N11—H11A	0.94 (3)
C20—N10	1.381 (2)	N12—H12A	0.94 (3)
			
C6—C1—C2	121.6 (2)	N11—C22—H22C	109.5
C6—C1—N1	118.9 (3)	H22A—C22—H22C	109.5
C2—C1—N1	119.5 (3)	H22B—C22—H22C	109.5
C1—C2—C3	118.3 (2)	N11—C23—H23A	109.5
C1—C2—H2	120.8	N11—C23—H23B	109.5
C3—C2—H2	120.8	H23A—C23—H23B	109.5
C2—C3—C4	124.5 (2)	N11—C23—H23C	109.5
C2—C3—N2	114.0 (2)	H23A—C23—H23C	109.5
C4—C3—N2	121.33 (18)	H23B—C23—H23C	109.5
C5—C4—C3	112.67 (17)	N12—C24—H24A	109.5
C5—C4—C7	123.22 (17)	N12—C24—H24B	109.5
C3—C4—C7	124.11 (17)	H24A—C24—H24B	109.5
C6—C5—C4	124.8 (2)	N12—C24—H24C	109.5
C6—C5—N3	113.30 (19)	H24A—C24—H24C	109.5
C4—C5—N3	121.83 (18)	H24B—C24—H24C	109.5
C1—C6—C5	118.0 (2)	N12—C25—H25A	109.5
C1—C6—H6	121.0	N12—C25—H25B	109.5
C5—C6—H6	121.0	H25A—C25—H25B	109.5
C8—C7—C10	120.10 (16)	N12—C25—H25C	109.5
C8—C7—C4	119.90 (15)	H25A—C25—H25C	109.5
C10—C7—C4	119.97 (16)	H25B—C25—H25C	109.5
O7—C8—N4	118.79 (16)	N12—C26—H26A	109.5
O7—C8—C7	124.11 (16)	N12—C26—H26B	109.5
N4—C8—C7	117.08 (15)	H26A—C26—H26B	109.5
O9—C9—N4	122.42 (17)	N12—C26—H26C	109.5
O9—C9—N5	122.07 (17)	H26A—C26—H26C	109.5
N4—C9—N5	115.51 (16)	H26B—C26—H26C	109.5
O8—C10—N5	118.72 (16)	O1—N1—O2	124.3 (3)
O8—C10—C7	124.59 (17)	O1—N1—C1	118.2 (3)
N5—C10—C7	116.68 (16)	O2—N1—C1	117.4 (3)
C16—C11—C12	121.82 (18)	O4—N2—O3	124.9 (2)
C16—C11—N6	119.5 (2)	O4—N2—C3	116.9 (2)
C12—C11—N6	118.7 (2)	O3—N2—C3	117.97 (19)
C13—C12—C11	117.63 (19)	O6—N3—O5	124.2 (2)
C13—C12—H12	121.2	O6—N3—C5	119.76 (17)
C11—C12—H12	121.2	O5—N3—C5	116.0 (2)
C12—C13—C14	124.84 (18)	C9—N4—C8	125.18 (16)
C12—C13—N7	113.99 (18)	C9—N4—H4	116.2 (14)
C14—C13—N7	121.06 (16)	C8—N4—H4	118.5 (14)
C15—C14—C13	112.90 (16)	C9—N5—C10	125.41 (16)
C15—C14—C17	123.66 (17)	C9—N5—H5	115.7 (16)
C13—C14—C17	123.42 (17)	C10—N5—H5	118.8 (16)
C16—C15—C14	124.62 (18)	O11—N6—O10	122.2 (2)
C16—C15—N8	114.02 (17)	O11—N6—C11	119.1 (2)
C14—C15—N8	121.13 (16)	O10—N6—C11	118.6 (2)
C11—C16—C15	117.95 (19)	O12—N7—O13	124.23 (18)
C11—C16—H16	121.0	O12—N7—C13	117.30 (17)
C15—C16—H16	121.0	O13—N7—C13	118.40 (17)
C18—C17—C20	120.41 (16)	O15—N8—O14	124.99 (18)
C18—C17—C14	119.19 (15)	O15—N8—C15	117.93 (16)
C20—C17—C14	120.34 (15)	O14—N8—C15	117.02 (17)
O16—C18—N9	118.05 (16)	C19—N9—C18	125.36 (16)
O16—C18—C17	125.23 (17)	C19—N9—H9	117.0 (15)
N9—C18—C17	116.70 (16)	C18—N9—H9	117.6 (15)
O18—C19—N10	122.54 (18)	C19—N10—C20	125.79 (16)
O18—C19—N9	122.14 (18)	C19—N10—H10	117.8 (16)
N10—C19—N9	115.32 (17)	C20—N10—H10	116.4 (16)
O17—C20—N10	118.43 (16)	C22—N11—C23	114.3 (3)
O17—C20—C17	125.15 (17)	C22—N11—C21	110.7 (2)
N10—C20—C17	116.42 (16)	C23—N11—C21	110.1 (2)
N11—C21—H21A	109.5	C22—N11—H11A	107 (2)
N11—C21—H21B	109.5	C23—N11—H11A	108.9 (19)
H21A—C21—H21B	109.5	C21—N11—H11A	105 (2)
N11—C21—H21C	109.5	C25—N12—C26	114.9 (3)
H21A—C21—H21C	109.5	C25—N12—C24	111.8 (3)
H21B—C21—H21C	109.5	C26—N12—C24	108.2 (3)
N11—C22—H22A	109.5	C25—N12—H12A	105.4 (18)
N11—C22—H22B	109.5	C26—N12—H12A	106.4 (19)
H22A—C22—H22B	109.5	C24—N12—H12A	109.8 (18)
			
C6—C1—C2—C3	−2.2 (3)	C20—C17—C18—O16	177.9 (2)
N1—C1—C2—C3	178.9 (2)	C14—C17—C18—O16	0.8 (3)
C1—C2—C3—C4	1.7 (3)	C20—C17—C18—N9	−0.2 (3)
C1—C2—C3—N2	−173.5 (2)	C14—C17—C18—N9	−177.35 (18)
C2—C3—C4—C5	0.6 (3)	C18—C17—C20—O17	179.4 (2)
N2—C3—C4—C5	175.41 (18)	C14—C17—C20—O17	−3.5 (3)
C2—C3—C4—C7	−179.3 (2)	C18—C17—C20—N10	0.2 (3)
N2—C3—C4—C7	−4.5 (3)	C14—C17—C20—N10	177.31 (18)
C3—C4—C5—C6	−2.6 (3)	C6—C1—N1—O1	−175.5 (3)
C7—C4—C5—C6	177.25 (19)	C2—C1—N1—O1	3.5 (4)
C3—C4—C5—N3	173.45 (18)	C6—C1—N1—O2	7.0 (4)
C7—C4—C5—N3	−6.7 (3)	C2—C1—N1—O2	−174.0 (3)
C2—C1—C6—C5	0.3 (3)	C2—C3—N2—O4	−49.6 (3)
N1—C1—C6—C5	179.3 (2)	C4—C3—N2—O4	135.1 (2)
C4—C5—C6—C1	2.3 (3)	C2—C3—N2—O3	125.8 (2)
N3—C5—C6—C1	−174.08 (19)	C4—C3—N2—O3	−49.6 (3)
C5—C4—C7—C8	136.11 (19)	C6—C5—N3—O6	135.8 (2)
C3—C4—C7—C8	−44.0 (3)	C4—C5—N3—O6	−40.7 (3)
C5—C4—C7—C10	−42.0 (3)	C6—C5—N3—O5	−41.6 (3)
C3—C4—C7—C10	137.9 (2)	C4—C5—N3—O5	141.9 (2)
C10—C7—C8—O7	179.62 (18)	O9—C9—N4—C8	178.88 (19)
C4—C7—C8—O7	1.5 (3)	N5—C9—N4—C8	−1.4 (3)
C10—C7—C8—N4	1.4 (3)	O7—C8—N4—C9	−178.87 (18)
C4—C7—C8—N4	−176.73 (17)	C7—C8—N4—C9	−0.5 (3)
C8—C7—C10—O8	178.54 (19)	O9—C9—N5—C10	−177.6 (2)
C4—C7—C10—O8	−3.4 (3)	N4—C9—N5—C10	2.7 (3)
C8—C7—C10—N5	−0.3 (3)	O8—C10—N5—C9	179.20 (19)
C4—C7—C10—N5	177.84 (17)	C7—C10—N5—C9	−1.9 (3)
C16—C11—C12—C13	−0.5 (3)	C16—C11—N6—O11	17.1 (4)
N6—C11—C12—C13	−177.93 (19)	C12—C11—N6—O11	−165.3 (3)
C11—C12—C13—C14	4.6 (3)	C16—C11—N6—O10	−161.4 (2)
C11—C12—C13—N7	−171.62 (18)	C12—C11—N6—O10	16.2 (3)
C12—C13—C14—C15	−4.4 (3)	C12—C13—N7—O12	−40.6 (2)
N7—C13—C14—C15	171.59 (16)	C14—C13—N7—O12	143.01 (19)
C12—C13—C14—C17	174.00 (18)	C12—C13—N7—O13	136.46 (19)
N7—C13—C14—C17	−10.0 (3)	C14—C13—N7—O13	−39.9 (3)
C13—C14—C15—C16	0.2 (3)	C16—C15—N8—O15	131.1 (2)
C17—C14—C15—C16	−178.20 (19)	C14—C15—N8—O15	−43.6 (3)
C13—C14—C15—N8	174.30 (16)	C16—C15—N8—O14	−46.3 (3)
C17—C14—C15—N8	−4.1 (3)	C14—C15—N8—O14	139.05 (19)
C12—C11—C16—C15	−3.3 (3)	O18—C19—N9—C18	−178.5 (2)
N6—C11—C16—C15	174.1 (2)	N10—C19—N9—C18	0.7 (3)
C14—C15—C16—C11	3.5 (3)	O16—C18—N9—C19	−178.6 (2)
N8—C15—C16—C11	−170.99 (18)	C17—C18—N9—C19	−0.3 (3)
C15—C14—C17—C18	132.5 (2)	O18—C19—N10—C20	178.5 (2)
C13—C14—C17—C18	−45.7 (3)	N9—C19—N10—C20	−0.7 (3)
C15—C14—C17—C20	−44.6 (3)	O17—C20—N10—C19	−178.9 (2)
C13—C14—C17—C20	137.12 (19)	C17—C20—N10—C19	0.3 (3)

### Hydrogen-bond geometry (Å, °)

**Table:**

*D*—H···*A*	*D*—H	H···*A*	*D*···*A*	*D*—H···*A*
N4—H4···O17^i^	0.85 (2)	1.94 (2)	2.780 (2)	170 (2)
N5—H5···O16	0.86 (3)	1.93 (3)	2.778 (2)	170 (2)
N9—H9···O8	0.85 (2)	1.95 (2)	2.790 (2)	172 (2)
N10—H10···O7^ii^	0.83 (2)	1.96 (2)	2.786 (2)	173 (2)
N11—H11A···O9^iii^	0.94 (3)	1.82 (3)	2.730 (2)	160 (3)
N12—H12A···O18	0.94 (3)	2.02 (3)	2.803 (3)	139 (3)
N12—H12A···O5^iv^	0.94 (3)	2.16 (3)	2.856 (3)	130 (2)
C2—H2···O4^v^	0.93	2.58	3.265 (3)	131.
C16—H16···O2^vi^	0.93	2.27	3.146 (3)	156.
C24—H24B···O5	0.96	2.51	3.448 (4)	164.
C24—H24C···O10^iii^	0.96	2.57	3.457 (4)	154.
C26—H26B···O18^iv^	0.96	2.50	3.400 (5)	156.

Symmetry codes: (i) *x*−1, *y*, *z*; (ii) *x*+1, *y*, *z*; (iii) *x*, −*y*+1/2, *z*+1/2; (iv) −*x*+1, −*y*, −*z*+1; (v) −*x*, −*y*, −*z*+2; (vi) −*x*+1, *y*+1/2, −*z*+3/2.
